# Interventions to reduce wait times for primary care appointments: a systematic review

**DOI:** 10.1186/s12913-017-2219-y

**Published:** 2017-04-20

**Authors:** Dominique Ansell, James A. G. Crispo, Benjamin Simard, Lise M. Bjerre

**Affiliations:** 1 Department of Family Medicine, University of Ottawa, Ottawa, ON Canada; 2 McLaughlin Centre for Population Health Risk Assessment, University of Ottawa, Ottawa, ON Canada; 3 Fulbright Canada Student, University of Pennsylvania, Philadelphia, PA USA; 4 Bruyère Research Institute, Ottawa, ON Canada; 5 School of Epidemiology, Public Health and Preventive Medicine, University of Ottawa, Ottawa, ON Canada; 6 Department of Family Medicine, C.T. Lamont Primary Health Care Research Centre, University of Ottawa, Ottawa, ON Canada

**Keywords:** Wait times, Primary care, Open access scheduling, Third next available appointment

## Abstract

**Background:**

Accessibility and availability are important characteristics of efficient and effective primary healthcare systems. Currently, timely access to a family physician is a concern in Canada. Adverse outcomes are associated with longer wait times for primary care appointments and often leave individuals to rely on urgent care. When wait times for appointments are too long patients may experience worse health outcomes and are often left to use emergency department resources. The primary objective of our study was to systematically review the literature to identify interventions designed to reduce wait times for primary care appointments. Secondary objectives were to assess patient satisfaction and reduction of no-show rates.

**Methods:**

We searched multiple databases, including: Medline via Ovid SP (1947 to present), Embase (from 1980 to present), PsychINFO (from 1806 to present), Cochrane Central Register of Controlled Trials (CENTRAL; all dates), Cumulative Index to Nursing and Allied Health (CINAHL; 1937 to present), and Pubmed (all dates) to identify studies that reported outcomes associated with interventions designed to reduce wait times for primary care appointments. Two independent reviewers assessed all identified studies for inclusion using pre-defined inclusion/exclusion criteria and a multi-level screening approach. Our study methods were guided by the Cochrane Handbook for Systematic Reviews of Interventions.

**Results:**

Our search identified 3,960 articles that were eligible for inclusion, eleven of which satisfied all inclusion/exclusion criteria. Data abstraction of included studies revealed that open access scheduling is the most commonly used intervention to reduce wait times for primary care appointments. Additionally, included studies demonstrated that dedicated telephone calls for follow-up consultation, presence of nurse practitioners on staff, nurse and general practitioner triage, and email consultations were effective at reducing wait times.

**Conclusions:**

To our knowledge, this is the first study to systematically review and identify interventions designed to reduce wait times for primary care appointments. Our findings suggest that open access scheduling and other patient-centred interventions may reduce wait times for primary care appointments. Our review may inform policy makers and family healthcare providers about interventions that are effective in offering timely access to primary healthcare.

**Electronic supplementary material:**

The online version of this article (doi:10.1186/s12913-017-2219-y) contains supplementary material, which is available to authorized users.

## Background

Timely access to primary healthcare is associated with improved health outcomes and contributes to cost control CFP Canada [1, 2]. Accessibility and availability are important characteristics of efficient and effective primary healthcare systems [3–5]. Canadians use ambulatory primary healthcare services for multiple reasons, including but not limited to routine care, mental health, minor but urgent health problems, child and maternity care, liaison with home care, health promotion and disease prevention, and end of life care. Having timely access to primary care has been shown to increase patient satisfaction as well as the quality of care delivered [5]. However, recent studies have demonstrated that access to family physicians in Canada is becoming increasingly difficult due to physician shortages and increasing rates of disability and chronic disease. In 2012, the Canadian Foundation for Healthcare Improvement reported that Canada had a higher percentage of sick adults (23%) who indicated waiting six or more days for a primary care appointment compared to Switzerland (2%) and New Zealand (5%) [6]. As such, wait times in healthcare are a growing concern for the Canadian population [7]. National surveys have also shown that Canadians struggle to schedule same-day appointments with their family physician and subsequently often rely on urgent care for non-urgent health issues [8].

Waiting for a primary healthcare appointment can often impose a physical and emotional burden on an individual who is in pain or worried about a serious health condition. Studies have shown that adverse consequences may arise from prolonged waiting for primary care appointments [9–11]. For example, prolonged wait times for cancer and heart disease have been associated with an increased risk of morbidity and mortality due to a delay in care [12–14]. Similarly, patients suffering from mental health conditions, who do not receive timely access to care, often experience a rapid decline in their condition and a lost opportunity for effective treatment [10]. Subsequently, worse health outcomes are often associated with higher costs to individuals, as waiting for care may be burdened by a loss of income due the inability to work [15]. Despite having one of the highest per capita spending for healthcare in the world, Canada often fails to offer timely access to primary care providers [3]. This is due to numerous factors such as a healthcare system that is governed at multiple levels, a lack of engagement of healthcare providers in a collaborative approach for policy development and implementation, and the need to implement measurement systems for quality improvement [16]. In Canada, the provincial government manages healthcare; however, several provinces have regional authorities that coordinate services, and certain costs [7, 17]. The regional health authorities are mandated to plan, fund and integrate hospital, home and community services, but often lack the authority to govern primary care services, which in turn limits their ability to improve access to primary care services. Moreover, despite the College of Family Physicians of Canada’s 2103 report on best practices to improve access to care, the multi-level governance of Canadian healthcare and variety of federal, provincial and territorial systems make it difficult to implement a collaborative approach [18].

To date, strategies designed to improve access to primary care have focused on increasing the supply of family physicians, implementing incentives for family physicians to take on additional or more complex patients, providing incentives for family physicians to increase the number of evening and weekend clinics they provide, and improving patient flow and practice management efficiency by using advanced access scheduling [1, 9]. One of the most economical techniques to improve access to primary care appointments has proven to be the use of open access scheduling [19, 20]. Open access scheduling focuses on reducing and eliminating delays without adding staffing resources [1]. Open access scheduling achieves this by keeping a majority of usually short appointment slots unscheduled, only filling them on the same day as patients call in for appointments. Conversely, longer or foreseeable routine appointments are scheduled ahead of time [8]. In turn, open access scheduling may help mitigate the continuous rise for chronic care appointments in primary care settings [21].

To improve primary care scheduling efficiency, several practices have implemented the use of the open access model, also known as same-day scheduling or advanced access. This scheduling model leaves approximately half of the day open, eliminating booking a physician’s schedule weeks and months in advance and keeping the rest of the day for clinically necessary follow-up appointments [22]. Compared to the traditional scheduling where the schedule is already full before the start of the workday, the open access model allows for more flexibility in scheduling, eliminates delays, and improves patient satisfaction and health outcomes [19, 23–25].

Extended family practice hours have been deemed efficient at reducing wait times [18, 26]. A significant proportion of family physicians in the US and in Canada now practice in group-based settings, which allows for after-hours coverage to be shared among physicians [27, 28]. In turn, this allows physicians to provide patients with more opportunities to be seen in a timely manner [29]. It has also been documented that patients who have access to evening and weekend care are less likely to visit the emergency department [30, 31]. Electronic communication and telephone follow-ups are other ways that primary care physicians can use which may reduce the number of unnecessary and time-consuming face-to-face appointments while maintaining continuity of care [10, 32]. When available, utilizing the medical expertise of the family physician, nurse, pharmacist, psychologist, social worker, dietician, and physiotherapist allow patients to be seen for various medical problems by the appropriate healthcare provider in a single physical setting. This allows physicians to more effectively care for their patients and subsequently leads to reduced wait times for primary care appointments [29, 33].

It is challenging for policy makers and care providers to make decisions pertaining to the implementation of interventions to reduce wait times for primary care appointments, as the literature on this topic has not yet been summarized. To address this gap we conducted a systematic review of the literature to identify interventions designed to reduce wait times for primary care appointments. Findings from this review may inform policy makers and health providers about options to reduce wait times for primary care appointments.

## Methods

All studies reporting outcomes associated with interventions to reduce wait times for primary care appointments were included in our review. Specifically, we included studies that 1) investigated an intervention in a primary care setting, 2) reported quantitative data on wait times for primary care appointments, and 3) compared intervention and nonintervention data, before-after comparisons in the same clinic, or an intervention in one setting compared to a similar setting without the same intervention. All study types, including randomized control trials, prospective studies, and retrospective studies meeting our inclusion criteria were included. Studies not identifying an intervention designed for the purpose of reducing wait times for primary care appointments were not included in this review. Commentaries, editorials, and narratives were excluded.

### Types of participants

Participants in this systematic review included care settings aiming to reduce wait times for primary care appointments.

### Types of interventions

Any intervention aimed at reducing wait times for primary care appointments compared to no intervention.

### Ethics

This study did not involve human participants; therefore, no ethics approval was required.

### Primary outcome

1. Wait time for primary care appointments.

### Secondary outcomes

1. Patient satisfaction.
2. Reduction in no-show rates.

### Search methods for identification of studies

Multiple databases were searched for articles on interventions to reduce wait times for primary care appointments. Databases included Medline, PubMed, Embase, the Cochrane Central Register of Controlled Trials (CENTRAL), the Cumulative Index to Nursing and Allied Health Literature (CINAHL) and PsycINFO. Search strategies were developed with the assistance of a Medical Librarian. Studies were restricted to those published in English or French. Search strategies are shown in Additional file 1.

### Data collection

The first level of screening was conducted by two review authors (DA and BS), who independently screened the titles and abstracts of all studies identified through literature searches. Studies deemed by both reviewers to not satisfy inclusion criteria were excluded, while all other studies advanced to full-text (second level) screening. For second level screening, DA and BS reviewed the full-text of remaining studies and assessed eligibility according to the pre-defined inclusion and exclusion criteria. Disagreements were resolved through discussion and with the input of an independent third party, as necessary.

### Data extraction and management

DistillerSR (Evidence Partners Ottawa, Canada) was used to complete screening and data abstraction. A standardized data abstraction form was tested prior to extracting data from included studies. Data was extracted on the items reported in Table 1 below. A descriptive approach was used to report the outcomes measured in the studies included in our systematic review, a meta-analysis was not performed.

**Table 1:** Data extraction items

Practice characteristics	Type of practice (family medicine academic center, primary care clinic, community primary care clinic, academic and hospital clinic, multispecialty group clinic)
Implementation	Type of intervention (s) implemented to reduce wait times
Comparator	Intervention in one setting compared to a similar setting without the intervention.
Outcomes	Measurement of wait times and comparison to previous wait times (before-after comparison, or comparison with control clinic). Measurement of patient satisfaction and no-show rates.

## Results

**Fig. 1:**
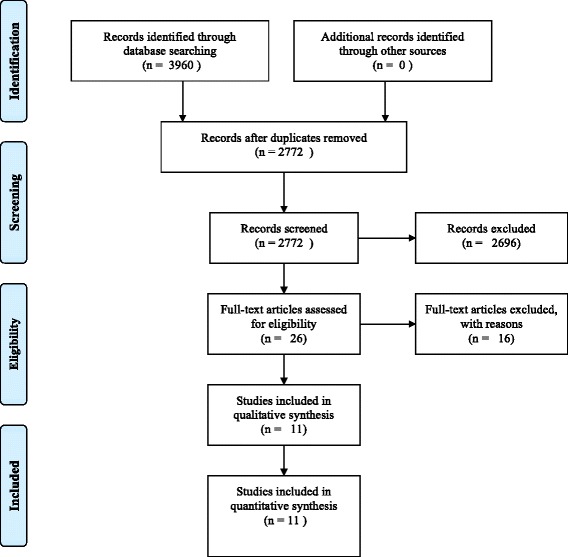
Prisma flow diagram of articles

Database searches yielded 3,960 articles published prior to January 22, 2015. A total of 2,722 articles remained after removal of duplicates and were screened according to pre-determined inclusion/exclusion criteria. Figure 1 displays a flow diagram of the selection process.

The overall weighted kappa, defined as the statistical measure of inter-rater agreement, was measured at 0.75 for the first level screening and 0.77 for the second level of screening [34]. Our kappa value indicates that the independent reviewers had excellent agreement regarding the inclusion/exclusion of studies.

Eleven studies satisfied our inclusion and exclusion criteria and were therefore included in our review (Table 2) [22, 30, 31, 35–42]. All eleven included studies were conducted in a primary care setting; however, three of the studies also incorporated multi-specialty groups or hospital clinics. All eleven studies reported using open access scheduling as an intervention to reduce wait times for primary care appointments. In addition to open access scheduling, Goodall et al. [37] and Pickin et al. [39] reported using nurse practitioners, telephone follow-up consultations, specific measures to reduce follow-up, general practitioner (GP) triage, nurse triage, redirecting workload from GPs, measures to promote self-care, and e-mail consultations as interventions to reduce wait times for primary care appointments (Table 3) [37, 39]. Lastly, Pickin et al. [39] showed open access scheduling combined with the use of telephone follow-ups, as well as the use of allied health professionals to improve appointment availability [39].

**Table 2:** Summary of included articles

Source	Primary Care	Trial Design	Type of clinic	Country of study	Type of intervention	# of intervention(s)in study	Improved wait time
Belardi et al. 2004 [30]	Yes	Controlled before-after	Family medicine academic centre	USA	Open access	1	Yes
Bundy et al. 2005 [35]	Yes	Uncontrolled before-after	Primary care clinics	USA	Open access	1	Yes
Cameron et al. 2010 [22]	Yes	Uncontrolled before-after	Family medicine academic clinics	Canada	Open access	1	Yes
Dixon et al. 2006 [36]	Yes	Uncontrolled before-after	Primary care clinics	United Kingdom	Open access	1	Yes
Goodall et al. 2006 [37]	Yes	Survey	Primary care clinics	United Kingdom	Multiple	8	Yes
Mehrota et al. 2008	Yes	Uncontrolled before-after	Community primary care clinics	USA	Open access	1	Yes
Parente et al. 2005 [38]	Yes	Uncontrolled before-after	Academic and hospital clinic	USA	Open access	1	Yes
Pickin et al. 2004 [39]	Yes	Survey	Primary care clinics of the National group	United Kingdom	Multiple	8	Yes
Salisbury et al. 2007 [40]	Yes	Survey	Primary care clinics	United Kingdom	Open access	1	Yes
Solberg et al. 2004 [41]	Yes	Uncontrolled before-after	Multispecialty group including primary care	USA	Open access	1	Yes
Sperl-Hillen et al. 2008 [42]	Yes	Uncontrolled before-after	Primary care clinics	USA	Open access	1	Yes

**Table 3:** Interventions used to reduce wait times in primary care

Intervention	Goodall et al. (*n* = 162) % using	Pickin et al. (*n* = 286) % using
Use of nurse practitioners	28.8%	10%
Telephone for follow-up consultation	66.2%	78%
Specific measures to reduce follow-up	76%	70%
GP triage	52%	41%
Nurse triage	58%	47%
Redirect workload from GP	75%	84%
Measure to promote self care	16%	64%
Email consultations	1%	7%

In all eleven included studies, the wait time prior to the implementation of an intervention was greater than after implementation (Table 4). The mean reduction in wait time for all included studies was 11.3 days (SD +/- 8.3 days). All studies reported the time to next appointment as the third next available appointment, defined as the number of days between the time when a patient makes a request for an appointment with a physician and the third available appointment for this patient [43]. This measure gives a more accurate reflection of true appointment availability by taking into account cancellations or other unexpected events [43].

**Table 4:** Time reduction for primary appointments pre and post intervention(s)

Source	Intervention	Wait time pre intervention (days)	Wait time post intervention (days)	Absolute reduction (Δ in days)
Belardi et al. 2004 [30]	Open access	21	4-7	14
Bundy et al. 2005 [35]	Open access	36	4	32
Cameron et al. 2010 [22]	Open access	13.7	3.6	10.1
Dixon et al. 2006 [36]	Open access	3.6	1.5	2.1
Goodall et al. 2006 [37]	Multiple^a^	24.3	10.2	14.1
Mehorta et al. 2008	Open access	21	11	10
Parente et al. 2005 [38]	Open access	18.7	11.8	6.9
Pickin et al. 2004 [39]	Multiple^a^	3.6	1.9	1.7
Sperl-HIllen et al. 2008 [42]	Open access	21.6	4.2	17.4
Salisbury et al. 2007 [40]	Open access	19.5	4.5	15
Solberg et al. 2004 [41]	Open access	17.8	4.2	13.6

^a^Multiple interventions included in the study were a combination of: open access, use of nurse practitioners, telephone for follow-up consultation, specific measures to reduce follow-up, GP triage, nurse triage, redirect workload from GP, email consultations

Three studies reported on patient satisfaction. Bundy et al. (2004) showed an increase in patient satisfaction post intervention, while Mehrotra et al. [31] and Parente et al. [38] did not show a significant improvement in patient satisfaction post intervention implementation (Table 5). No-show rates were reported in four studies and were shown to decrease following implementation of the intervention (Table 6). Other outcomes measured in included studies were continuity of care, physician satisfaction, and urgent care utilization. Each of these outcomes was only studied in one of the eleven articles identified for data abstraction; therefore, they were not further summarized.

**Table 5:** Measurement of patient satisfaction with pre and post intervention

Source	Intervention	Patient satisfaction pre intervention	Patient satisfaction post intervention	Absolute reduction (Δ in %)
Bundy et al. 2004	Open access	45%	61%	-16%
Mehrotra et al. 2008 [31]	Open access	53%	51%	2%
Parente et al. 2005 [35]	Open access	89%	87%	2%

**Table 6:** Percentage of no-show rates pre and post intervention in primary care

Source	Intervention	No show rate pre -intervention	No show rate post-intervention	Absolute reduction (Δ in %)
Belardi et al. 2004 [30]	Open access	8.6%	6.7%	1.9
Bundy et al. 2005 [35]	Open access	16%	11%	5
Cameron et al. 2010 [22]	Open access	3.33%	1.89%	1.44
Mehrota et al. 2008	Open access	14%	14%	0

## Discussion

It has been reported that patients are more satisfied when wait times for primary care appointments are minimal or non-existent [27]. Moreover, access to primary care appointments has been shown to play a crucial role in reducing mortality rates [15]. Interventions shown to increase the availability of primary care appointments include open access scheduling, the use of nurse practitioners, telephone calls for follow-up consultations, and redirecting general practitioner workload [37]. Our systematic review identified eleven articles satisfying our inclusion criteria, which described a variety of interventions aimed at reducing wait times for primary care appointments. Open access scheduling was reported by all eleven studies as an effective intervention at reducing wait times.

### Open access scheduling

Open access scheduling is based on several principles that favor access to care. It focuses on understanding demand and matching capacity. As demonstrated by our review, several primary care practices have implemented this system and have achieved reduced wait times [22, 30, 31, 35–42]. However, the implementation of open access scheduling can prove to be challenging in certain settings. In a study by Pickin et al. [39] it was demonstrated that the increasing number of same-day appointments proved to be preferred by some patient populations (mostly the younger patient), but less preferred by an older patient population who seemed to favour pre-booked appointments [39]. This study, among others, suggested open access scheduling as an ideal way to accommodate the needs and preferences of multiple populations, which is consistent with patient-centered approaches to the delivery of health services [39].

### Telephone consultations

In two of the included studies, primary care practices implemented the use of telephone calls for consultations or follow-up with favorable results and demonstrated combined effects to the reduction in wait times in with open access scheduling [39, 40]. In the study by Pickin et al. [39], telephone consultation was often reported as a successful intervention to reduce wait times for primary care appointments. Additionally, telephone consultation has been shown to be beneficial due to its ability to improve public access to medical information, and ensuring adequate follow-ups for individuals affected from chronic care conditions [44]. By offering more efficient methods of communication, unnecessary appointments can be eliminated, which in turn enables additional patients, in a given doctor’s practice, to see their primary care physician in person [44–46].

### Primary teams

The use of teams in primary care practices enables individuals to experience more timely access to clinicians. In our review, two studies identified the use of nurse practitioners and allied workers as an approach to increase practice efficiency. Additionally, the use of allied health professionals, with open access scheduling, has been shown to have a combined effect at reducing wait times in primary care [39]. Highly skilled nurses may provide more services for certain patient populations. For these reasons, the team-based approach to the provision of healthcare is being adopted by some primary care practices [37, 39].

### Secondary outcomes

In four of the included studies, the rate of patients who did not show-up for their appointment before the intervention was measured and compared to the rate of no-show appointments post-intervention [22, 30, 31, 35]. No-show rates decreased after the implementation of open access scheduling in each study, with a mean reduction in no-show rates of 2.78%. Patients who failed to show-up for their medical appointments had a negative impact on the productivity of the clinic as they take up a slot for other patients who are waiting for care. One study reported that no-show rates declined with open access scheduling because patients were more likely to be scheduled with their family physician [30]. Patient satisfaction only increased in one study, which could be attributed to the fact that a considerable proportion of patients prefer to schedule an appointment at a convenient time rather than having quick access to care. Other outcomes examined in the included studies were: the management of diabetes, the effect on use of urgent care, and physician satisfaction with the intervention [31, 41]. Sperl-Hillen et al. [42] evaluated the use of open access on the quality of care of diabetic individuals, and reported an increase in patients achieving optimal hemoglobin A1C levels as well as low-density liproprotein control (32). Solberg et al. [41] reported on the number of urgent care carry-over appointments, defined as urgent care referrals made for individuals who could not be accommodated with timely appointments at the clinic. The study reported a reduction of 5000 urgent care appointments 1 year after the implementation of open access scheduling (22). Lastly, Mehrotra et al. [31] reported on physician satisfaction with open access scheduling, showing a 24% increase in the proportion of staff who felt access to appointments was ‘very good’ or ‘excellent’ in comparison to access prior to open access scheduling (31, [47]. Our study was not designed to report on the effect of interventions on practice revenue and cost; however, it should be noted that future studies should look at the cost of implementing interventions to reduce wait times as secondary outcomes. The implementation of interventions to reduce wait times have costs, but such costs are countered by the savings yielded by decreasing the number of no-shows, improving the ability of physicians to see their own patients and providing more efficient services during the visit [18, 42, 48].

Systematic reviews provides a critical assessment and summary of the available literature of a given research question. As such, our study offers a thorough evaluation of studies that identify interventions designed to reduce wait times for primary care appointments. We ran searches in multiple databases in effort to identify all studies that satisfied our inclusion criteria. Our included studies reported evidence from Canada, the USA, and the United Kingdom. It should be noted that studies on interventions to reduce wait times for primary care appointments from Europe and Australia were identified in by our systematic search of the literature; however, identified studies did not meet our explicit inclusion criteria. Several countries have implemented innovative information systems, healthcare teams, and programs to improve chronic care patient outcomes [49].

All retrieved articles were systematically appraised using a double-blinded reviewer process. We used a web-based tool to assure quality and a systematic approach to data collection. Study limitations include: 1) we did not search grey literature, including conference proceedings, meeting abstracts, or government, and professional organizational websites; and 2) we did not contact authors of existing studies or other experts in the field to inquire about unpublished interventions to reduce wait times for primary care appointments, and 3) we only included studies published in English or French. These limitations could have prevented us from identifying additional studies reporting on interventions designed to reduce wait times for primary care appointments.

## Conclusions

Increasing demands on primary care, and issues of access to care continue to be relevant and sometimes contentious topics of discussion for individuals seeking care, their physicians, and policy makers. [31]. Our study systematically reviewed and summarized the literature on interventions designed to reduce wait times for primary care appointments. We found that open access scheduling significantly reduced wait times and is the most widely implemented intervention to reduce wait times in primary care settings. Future research should be directed at measuring the cumulative effect of interventions to reduce wait times for primary care appointments in order to explore interactions between open access and other interventions to reduce wait times. Subsequent research should also focus on practice cost and revenue and include a cost benefit analyses to the implementation of interventions. Lastly, future studies should also be directed at measuring the impact of interventions on the quality of care, the continuity of care and the impact on other healthcare services utilization.

## Additional file

Additional file 1: Search strategies: contains all search strategies for each database. (DOCX 17 kb)

## Abbreviations

CENTRAL: Cochrane central register of controlled trials
CINAHL: Cumulative index to nursing and allied health literature
GP: General practitioner
