# Supplementary material for: Interventions to reduce wait times for primary care appointments: a systematic review
Source: BMC Health Serv Res. 2017 Apr 20;17:295. doi: 10.1186/s12913-017-2219-y (PMC5397774; doi:10.1186/s12913-017-2219-y)
Supplement: Additional file 1: — Search strategies: contains all search strategies for each database. (DOCX 17 kb) [file 12913_2017_2219_MOESM1_ESM.docx]

**Search strategies**

**Additional file 1.**

**Medline Search strategy via Ovid SP (to be modified for the search of other databases):**

1. exp Family Practice/
2. exp Physicians, Family/
3. exp Primary Health Care/
4. (famil* adj2 practic*).tw.
5. (primary adj2 health adj2 care).tw.
6. (primary adj2 care).tw.
7. (famil* adj2 physician*).tw.
8. practionner*.tw.
9. (general adj2 practice*).tw.
10. OR 1/9
11. exp Appointments and Schedules/
12. schedul*.tw.
13. (priorit* adj2 score*).tw.
14. (third adj2 next adj2 availab* adj2 appointment*).tw.
15. (advance* adj2 access* adj2 model*).tw.
16. (same-day* adj2 access*).tw.
17. (open* adj2 access* adj2 model*).tw.
18. (carve-out adj2 model*).tw.
19. (access* adj2 model*).tw.
20. OR/ 11-19
21. “Health-Services Accessibility”/
22. wait*.tw.
23. time*.tw.
24. (service* adj2 accessib*).tw.
25. OR/ 21-24
26. 10 AND 20 AND 25

**Additional file 1.**

**Embase (from 1980 to present) search strategy via Ovid SP**

1. Family Practice.mp. or general practice/

2. exp physicians, Family/

3. exp Family Practice/

4. exp Primary Health Care/

5. (famil* adj2 practic*).tw.

6. (primary adj2 health adj2 care).tw.

7. (primary adj2 care).tw.

8. (famil* adj2 physician*).tw.

9. practitionner*.tw.

10. (general adj2 practice*).tw.

11. OR/ 1 or 2 or 3 or 4 or 5 or 6 or 7 or 8 or 9 or 10

12. exp appointments/ and schedules.mp. [mp=title, abstract, subject headings, heading word, drug trade name, original title, device manufacturer, drug manufacturer, device trade name, keyword]

13. schedul*.tw.

14. (priorit* adj2 score*).tw.

15. (third adj2 next adj2 availabl* adj2 appointment*).tw.

16. (same-day* adj2 access*).tw.

17. (open* adj2 access adj2 model*).tw.

18. (carve-out adj2 model*).tw.

19. (access* adj2 model*).tw.

20. OR/ 12 or 13 or 14 or 15 or 16 or 17 or 18 or 19

21. exp health care delivery/

22. time*.tw.

23. wait*.tw.

24. (service* adj2 accessib*).tw.

25. (health adj2 care adj2 accessib*).tw.

26. OR/ 21 or 22 or 23 or 24 or 25

27. 11 AND 20 AND 26

**Additional file 1**

**PsychINFO (from 1806 to present) search strategy via Ovid SP**

1. exp Family Physicians/

2. exp Family Medicine/

3. exp Primary Health Care/

4. (famil* adj2 practic*).tw.

5. (primary adj2 health adj2 care).tw.

6. (family adj2 medicine*).tw.

7. (primary adj2 care).tw.

8. (famil* adj2 physician*).tw.

9. practionner*.tw.

10. (general adj2 practice*).tw.

11. OR/ 1 or 2 or 3 or 4 or 5 or 6 or 7 or 8 or 9 or 10

12. (appointments and schedules).mp. [mp=title, abstract, heading word, table of contents, key concepts, original title, tests & measures]

13. schedul*.tw.

14. (priorit* adj2 score*).tw.

15. (third adj2 next adj2 availabl* adj2 appointment*).tw.

16. (advance* adj2 access* adj2 model*).tw.

17. (same-day* adj2 access*).tw.

18. (open* adj2 access adj2 model*).tw.

19. (carve-out adj2 model*).tw.

20. (access* adj2 model*).tw.

21. OR/ 12 or 13 or 14 or 15 or 16 or 17 or 18 or 19 or 20

22. exp Health Care Services/

23. exp Health Care Delivery/

24. wait*.tw.

25. time*.tw.

26. Service accessibility.mp.

27. (service adj2 accessib*).tw.

28. (health adj2 care adj2 deliver*).tw.

29. (health adj2 care adj2 service*).tw.

30. OR/ 21 or 22 or 23 or 24 or 25 or 26 or 27 or 28

31. 11 AND 20 AND 30

**Additional file 1**

**Cochrane Central Register of Controlled Trials (CENTRAL; all dates) search via Ovid SP**

1. exp Family Practice/

2. exp Physicians, Family/

3. Primary Health Care/

4. (famil* adj2 practic*).tw.

5. (primary adj2 health adj2 care).tw.

6. (primary adj2 care).tw.

7. (famil* adj2 physician).tw.

8. (general adj2 practice*).tw.

9. OR/ 1 or 2 or 3 or 4 or 5 or 6 or 7 or 8

10. exp "Appointments and Schedules"/

11. schedul*.tw.

12. (same-day* adj2 access*).tw.

13. (carve-out adj2 model*).tw.

14. (access* adj2 model*).tw.

15. (third adj2 next adj2 availabl* adj2 appointment*).tw.

16. OR/ 10 or 11 or 12 or 13 or 14 or 15

17. exp Health Services Accessibility/

18. wait*.tw.

19. time*.tw.

20. (service* adj2 accessib*).tw.

21. (health adj2 care adj2 accessib*).tw.

22. OR/16 or 17 or 18 or 19 or 20 or 21

22. 9 AND 15 AND 21

**Additional file 1**

**Cumulative Index to Nursing and Allied Health (CINAHL; all dates)**

1. (MH “Family medicine”)

2. Family medicine

3. (MH “Primary Health care”)

4. Primary health care

5. (MH “Physicians, Family)

6. Family physicians

7. (MH “Family practice”)

8. Family practice

9. OR/ 1 to 8

10. (MH “Appointments and Schedules”)

11. (MH “Appointment and Scheduling Information Systems)

12. Advanced access*

13. Third next available appointment*

14. Carve-out model*

15. Access model*

16. OR 10 to 15

17. Time*

18. Wait*

19. (MH “Health Services Accessibility”)

20. OR 17 to 19

21. 9 AND 16 AND 20

**Additional file 1**

PubMed (all dates)

("General practitioners"[MeSH Terms] OR "Physicians, Family"[MeSH Terms] OR "Primary health care"[MeSH Terms] OR "Family practice"[MeSH Terms])

AND

("Appointments and Schedules"[MeSH Terms] OR "advanced access*"[Text Word] OR "access model*"[Text Word] OR "open access*"[Text Word] OR "same-day access*"[Text Word])

AND

("Health Services Accessibility"[MeSH] OR "wait*"[Text Word] OR "time*"[Text Word])
